# Chick Embryo Chorioallantoic Membrane as a Platform for Assessing the In Vivo Efficacy of Chimeric Antigen Receptor T-cell Therapy in Solid Tumors

**DOI:** 10.4049/immunohorizons.2400059

**Published:** 2024-08-30

**Authors:** Allison J. Nipper, Emilie A. K. Warren, Kershena S. Liao, Hsuan-Chen Liu, Chieko Michikawa, Caroline E. Porter, Gabrielle A. Wells, Mariana Villanueva, Fabio Henrique Brasil da Costa, Ratna Veeramachaneni, Hugo Villanueva, Masataka Suzuki, Andrew G. Sikora

**Affiliations:** * Department of Head and Neck Surgery, University of Texas M.D. Anderson Cancer Center, Houston, TX; † Department of Otolaryngology-Head and Neck Surgery, Baylor College of Medicine, Houston, TX; ‡ Department of Family and Community Medicine, Baylor College of Medicine, Houston, TX; § Center for Cell and Gene Therapy, Baylor College of Medicine, Houston, TX; ¶ Advanced Technology Cores, Baylor College of Medicine, Houston, TX; ǁ Texas Children’s Hospital, Houston, TX; # Dan L. Duncan Comprehensive Cancer Center, Baylor College of Medicine, Houston, TX

## Abstract

The fertilized chicken egg chorioallantoic membrane (CAM), a highly vascularized membrane nourishing the developing embryo, also supports rapid growth of three-dimensional vascularized tumors from engrafted cells and tumor explants. Because murine xenograft models suffer limitations of time, cost, and scalability, we propose CAM tumors as a rapid, efficient screening tool for assessing anti-tumor efficacy of chimeric Ag receptor (CAR) T cells against solid tumors. We tested the efficacy of human epidermal growth factor receptor 2 (HER2)–specific CAR T cells against luminescent, HER2-expressing (FaDu, SCC-47) or HER2-negative (MDA-MB-468) CAM-engrafted tumors. Three days after tumor engraftment, HER2-specific CAR T cells were applied to tumors grown on the CAM. Four days post–CAR T cell treatment, HER2-expressing FaDu and SCC-47 tumors treated with CAR T showed reduced viable cancer cells as assessed by luciferase activity. This reduction in viable tumor cells was confirmed by histology, with lower Ki-67 staining observed in CAR T cell–treated tumors relative to T cell–treated controls. Persistence of CAR T in CAM and tumor tissue 4 days post-treatment was confirmed by CD3 staining. Altogether, our findings support further development of the chick CAM as an in vivo system for rapid, scalable screening of CAR T cell efficacy against human solid tumors.

## Introduction

Although adoptive cellular therapy with chimeric Ag receptor (CAR) T cells has achieved great results in hematologic cancers (1, 2), the application of CAR T to solid tumors lags behind the durable remissions and even cures of disease seen in liquid tumors (3, 4; reviewed in Refs. 5 and 6). Solid tumors present many unique obstacles to the CAR T cell strategy, including the immunosuppressive tumor microenvironment and the need for CAR T cells to infiltrate solid tumors through their aberrant vasculature (reviewed in Ref. 7 and 8). Additionally, CAR T cells must target cell surface–expressed Ags, which requires the identification of a suitable tumor-associated Ag; however, no universally effective target Ags have been identified for solid tumors. Because of the tremendous diversity of potential Ags in solid tumors, identification of suitable CAR T cell targets requires rigorous preclinical testing of potential candidates in the tumor type of interest (9). This preclinical testing can be a lengthy and costly process, often using cell line–derived xenografts grown in immunocompromised mouse models. These methods are not readily scalable for screening and preclinical validation due to their complexity and expense. As the targeting strategies of CAR T cells become increasingly intricate (reviewed in Ref. 10), there is greater need for development of rapid and economical preclinical screening methods for identification of the most promising CAR T cell candidates to pursue with further testing and clinical investigation.

In this article, we establish a methodology for modeling treatment of solid tumors with CAR T cells in the chicken egg chorioallantoic membrane (CAM) model system. Since 1913, the CAM has served as an in vivo culture system to study three-dimensional tumor growth, angiogenesis, and metastasis (11; reviewed in Ref. 12). The CAM is a highly vascularized membrane below the shell of fertilized chicken eggs that transports oxygen, nutrients, and calcium to the developing chick. This capacity for transport of gases and metabolites creates an environment that supports the growth of host and engrafted tissue alike, resulting in development of three-dimensional in vivo tumor grafts. Growth of CAM-engrafted tumors occurs relatively quickly, and xenografts can be seeded, treated, and harvested on the CAM model within 7–10 d. As a rapid and inexpensive system for growth of vascularized three-dimensional tumors, CAM tumor models offer significant advantages to assess CAR T cell efficacy against human solid tumors in vivo.

Among the myriad tumor-associated Ags being investigated as potential CAR T cell targets, human epidermal growth factor receptor 2 (HER2) stands out as a versatile candidate due to its expression in many solid tumors, including breast, colon, and head and neck squamous cell carcinoma (13–15). Using clinically tested HER2 CAR T cells (16), we demonstrate in this article that HER2-specific CAR T cell treatment of CAM-grown, HER2-expressing solid tumors reduced the presence of viable tumor cells and Ki-67 staining in tumors. Following CAR T cell treatment, we observe stable CD3^+^ immunostaining within tumor tissue, demonstrating persistence of transferred T cells and proximity to the tumor graft. Together, these data support development of the chicken egg CAM as a model system for rapid screening of the anti-tumor efficacy of CAR T cells in vivo.

## Materials and Methods

### Cell culture and characterization of HER2 expression

The human oropharyngeal carcinoma cell line FaDu and the human breast adenocarcinoma cell line MDA-MB-468 were obtained from the American Type Culture Collection (Manassas, VA). Human oral cavity carcinoma cell line SCC-47 was obtained from EMD Millipore (Burlington, MA). To generate cell lines expressing EGFP-*ffLuc* (firefly luciferase), the cells were infected with retrovirus encoding EGFP-*ffLuc* as previously described (17, 18).

All three cell lines were maintained in DMEM high glucose growth medium (Sigma, St. Louis, MO) supplemented with 10% FBS (Sigma) and 1% penicillin/streptomycin (Life Technologies, Gaithersburg, MD) and cultured under recommended conditions with regular mycoplasma testing. Flow cytometry was used to verify HER2-receptor expression, using an anti-HER2 Ab (29D8) (Cell Signaling Technologies, Beverly, MA).

### CAM preparation and tumor seeding

All CAM culture procedures, including humane euthanasia of chicken eggs, were carried out according to the 2020 guidelines of the American Veterinary Medical Association (19); per institutional and National Institutes of Health Office of Laboratory Animal Welfare requirements (20), an institutional animal care and use committee protocol was not required. Specific pathogen-free fertilized chicken eggs (embryonic day 0) (Charles River, North Franklin, CT) were placed in a humidified rocking incubator at 37°C on the day of arrival, for an initial period of 7 d. All subsequent manipulation of the eggs occurred inside a laminar flow hood. The chicken egg CAM was prepared according to our established protocols (12, 21, 22). The inner shell membrane was first removed using fine-point forceps to expose the CAM. Clear tape was used to cover the window in the shell, and the eggs were allowed to rest for 1 h at 37°C in a humidified Styrofoam incubator. During this time, tumor cells were harvested from tissue culture flasks and resuspended at a concentration of 2 million cells/60 µl in PBS^+/+^ (Life Technologies) for SCC-47 and FaDu and 2.5 million cells/60 µl for MDA-MB-468. Matrigel basement membrane matrix (Corning, Corning, NY) was added to the cell suspensions, bringing the total volume to 100 µl per egg. A silicone ring (Corning) was placed onto the exposed CAM of each egg, and 100 µl of the cell suspension–Matrigel mixture was pipetted into the center of the ring. The eggs were then returned to the humidified, temperature-controlled Styrofoam incubators for the remainder of the experiment. Every day, the eggs were examined for viability; any dead eggs were removed and disposed of accordingly.

### HER2-specific CAR T cells

Blood was obtained with consent from healthy donors under the local Institutional Review Board approved protocols. Human PBMCs were isolated using Ficoll-Paque Plus according to the manufacturer’s instructions (Axis-Shield, Dundee, UK). The vector encoding the HER2-directed CAR incorporating the CD28 costimulatory endodomain (second-generation HER2.CD28.ζCAR) (23–26), and the methodology to produce retrovirus and CAR T cells have been described previously (26). Briefly, PBMCs were activated with OKT3 (1 mg/ml) (Ortho Biotech, Raritan, NJ) and CD28 Abs (1 mg/ml) (Becton Dickson in RPMI-1640 (Hyclone) complete medium supplemented with 0.5% GlutaMAX (100×) (Life Technologies, Franklin Lakes, NJ) and fed every 2 days, beginning the day after stimulation, with medium supplemented with 10 ng/ml each recombinant human IL-7 and IL-15 (Peprotech). On day 2 post-OKT3/CD28 T blast generation, activated T cells (0.125 × 10^6^/ml) were added to HER2-directed CAR retroviral-coated plates and centrifuged at 400*g* for 5 min.

### In vivo imaging and tumor treatment

On day 3 postengraftment, the eggs were removed from the Styrofoam incubators and randomly assigned control T cell treatment or HER2-specific CAR T cell treatment groups. In situ luminescence of the tumors was measured with the IVIS Lumina III *In Vivo* imaging system (Perkin Elmer, Waltham, MA) after addition of 100 µl of 15 mg/ml d-luciferin (Gold Bio, St. Louis, MO) in PBS. CAR T cells were harvested from culture and resuspended in 75 µl of T cell growth medium at a ratio of 1:10 effector-to-tumor ratio (E:T). For example, for the 2 million FaDu cells engrafted onto each CAM, 200,000 CAR T cells were prepared in a 75-µl volume. A pipettor was used to slowly apply the CAR T cell suspension to the growing tumor within the silicone ring. The control group received 75 µl of expanded, nontransduced T cells. After addition of treatment or control cells, the eggs were returned to their Styrofoam incubators. On day 7 postengraftment, the eggs were placed at 4°C for a minimum of 30 min to anesthetize prior to removal of the shell around the original window for IVIS and brightfield imaging.

### Tumor harvest and immunohistochemistry

Following imaging on day 7, representative tumors were processed and stained for immunohistochemistry (IHC). Each tumor was excised from the CAM using forceps and scissors. These were harvested and fixed in 10% formalin at 4°C. After 24–48 h, the formalin was replaced with 70% ethanol. The formalin-fixed tissues were submitted in cassettes to the Baylor College of Medicine Pathology and Histology Core Laboratory and the M.D. Anderson Research Histology Core Laboratory for paraffin embedding and sectioning, as well as staining for H&E, pan-cytokeratin (Novus Biologicals, Littleton, CO), Ki-67 (Cell Signaling Technology, Danvers, PA), HER2 (Roche, Indianapolis, IN), and CD3 (Santa Cruz Biotechnology Dallas, TX). The tumors selected for CD3 staining were treated with E:T ratios of 1:1 or 1:2.5. The slides were imaged with the Aperio ScanScope (Aperio, Deer Park, IL) at 40×. Aperio ImageScope slide viewing software (Aperio) was used for image capture.

### Statistical analysis

Data analysis and graph creation were performed using Prism version 9 (GraphPad, San Diego, CA), and statistical comparisons between two timepoints or treatment groups were performed using the Mann–Whitney *U* test. For IHC quantification, QuPath (27) Positive Cell Detection and H-score analysis were performed on three representative regions at 20× per slide. Intensity parameters for threshold and background were consistent across all regions.

## Results

### Establishment of HER2-expressing tumor models on the CAM

**FIGURE 1.**
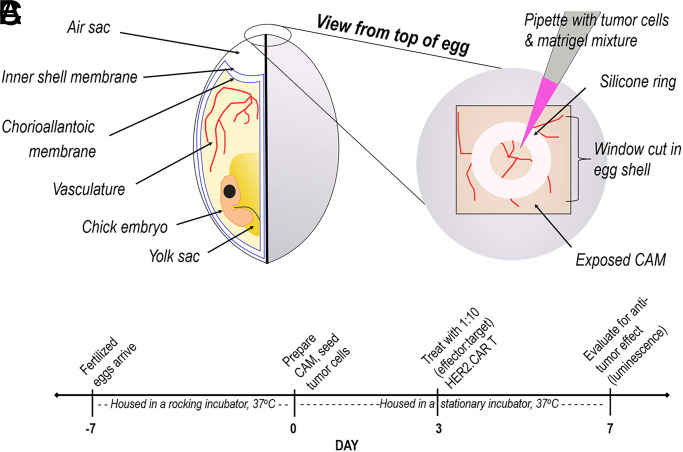
Preparation and CAR T cell treatment of the chicken egg chorioallantoic membrane. (**A**) Chicken egg and CAM anatomy. (**B**) Preparation methodology for tumor cell line engraftment on the CAM. (**C**) Experimental timeline.

**FIGURE 2.**
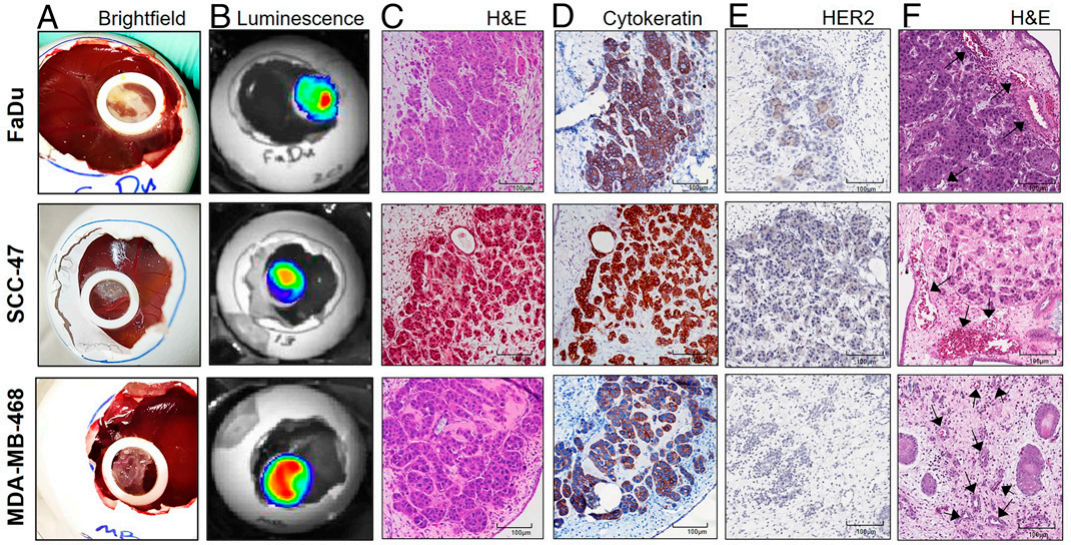
Establishment of tumor models on the CAM. Representative imaging of CAM-grown tumors harvested 7 days after engraftment of tumor cells. (**A–F**) Characteristics of tumors demonstrated in gross image (A), in vivo luminescence imaging (B), and representative tumor histology demonstrating tumor growth (C and D), HER2 expression (E), and location of vasculature (F) with H&E (C and F) and cytokeratin (D) staining. Scale bar, 100 microns.

To establish our CAM model of CAR T cell treatment (Fig. 1), we first confirmed growth of solid tumors from target cell lines on the CAM. HER2-expressing head and neck squamous cell carcinoma cell lines FaDu and SCC-47 served as positive controls expressing the target Ag for HER2-specific CAR T cell anti-tumor activity, whereas the breast adenocarcinoma cell line MDA-MB-468, which does not express HER2, served as a negative control. HER2 status of these cell lines has been previously reported by our group (Shaw) and was reconfirmed by flow cytometry (data not shown) and IHC (Fig. 2E). All three cell lines also expressed the *ffLuc* gene, allowing use of luciferase activity to quantify relative tumor burden. Seven days after tumor cells were engrafted on the CAM, the tumors were well established both on visual gross examination and by luminescence (Fig. 2A, 2B). The ability of each cell line to establish tumors on the CAM was confirmed by H&E and cytokeratin IHC staining (Fig. 2C, 2D). Vascularization of CAM tissue surrounding tumors was confirmed by histology (Fig. 2F).

### CAR T cells infiltrate tumors grown on the CAM

**FIGURE 3.**
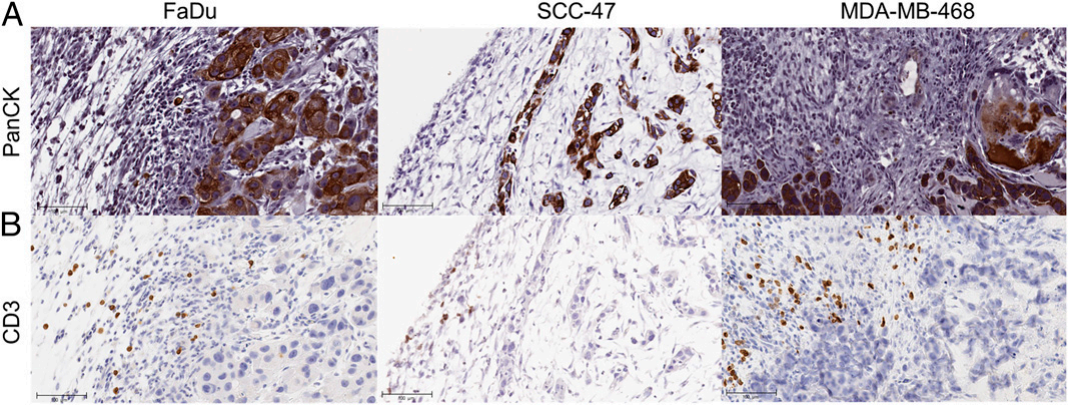
CAR T cell infiltration of CAM tissue. (**A**) Pan-cytokeratin staining of CAM tumors (PanCK). (**B**) CAR T cell detection in CAM tissue (CD3). The issues were collected on day 7. Scale bar, 100 microns.

To examine CAR T cell infiltration of and localization within tumors grown on the CAM, a separate set of CAM tumors in FaDu, SCC-47, and MDA-MB-468 were harvested for IHC staining. These tumors were treated with an increased E:T ratio of 1:2.5 to enhance the visibility of CAR T cells against the background of tumor tissue. Anti-CD3 Ab, a pan-T cell marker, was used to identify CAR T cells. Areas of tumor and T cell infiltrates were identified by examining the pan-cytokeratin staining (Fig. 3A). CD3-positive CAR T cells are clearly identified within the tissue sections (Fig. 3B), demonstrating their persistence after inoculation. However, we observed for both HER2-positive FaDu tumors and HER2-negative MDA-MB-468 tumors; the majority of CAR T cells accumulate at the interface of epithelial tumor nests with CAM stroma.

### HER2-targeting CAR T cells demonstrate Ag-dependent killing of cell lines on the CAM

**FIGURE 4.**
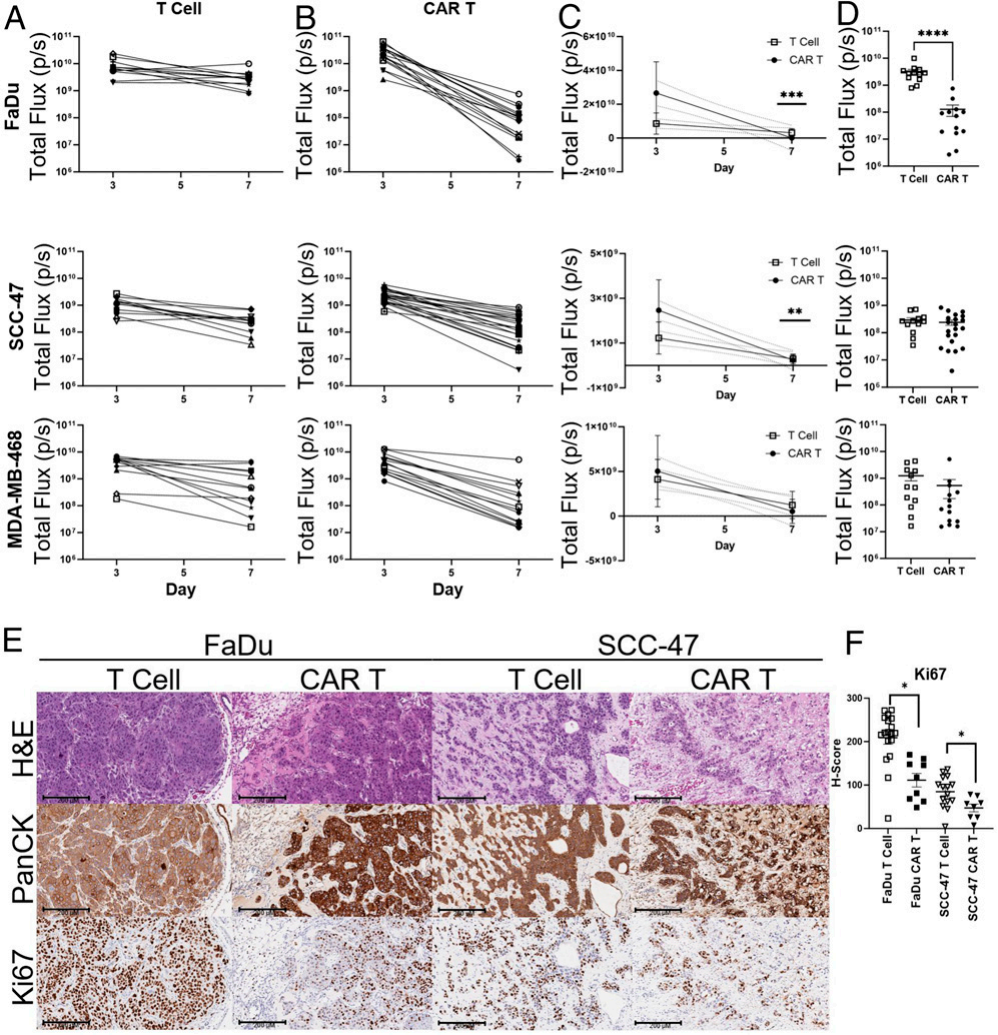
Quantification of tumor growth on the CAM following CAR T cell treatment. (**A** and **B**) Time course of luciferase activity of CAM tumors on experimental days 3 and 7 captured by IVIS imaging in T cell (A) or CAR T cell–treated (B) tumors. (**C**) Linear regression of T cell and CAR T cell–treated tumors, with statistical comparison of slopes. ***p = 0.0005, **p = 0.004. (**D**) Day 7 luciferase activity by IVIS imaging. ****p < 0.0001. (**E**) Representative regions of interest of pan-cytokeratin (PanCK) and Ki-67 staining on day 7 in T cell or CAR T cell–treated eggs. Scale bar, 200 microns. (**F**) Quantification of Ki-67 staining of CAM tumors by H-Score using Qupath. Three regions of interest selected per slide. *p < 0.05.

Once growth of cell lines on the CAM was established, we examined Ag specific killing by CAR T cells in CAM-grown tumors. Day 3 postengraftment, the tumors were treated with HER2-specific CAR T or control T cells to assess Ag specific killing of CAM tumor by CAR T cells. IVIS imaging of tumor luminescence allowed for detailed observation of the changes in tumor size, both immediately before T cell (Fig. 4A) or CAR T cell (Fig. 4B) application (day 3) and after treatment (day 7). This corresponded to a significant difference in the slope of growth patterns for CAR T cell– versus T cell–treated tumors from FaDu and SCC-47 cell lines, but not MDA-MB-468 (Fig. 4C). Most dramatically, luminescence of viable tumor cells was significantly reduced by day 7 in CAR T cell–treated FaDu tumors (Fig. 4D). Growth of tumor cells following treatment was visually confirmed by IHC staining of pan-cytokeratin (Fig. 4E). To assess proliferation of CAM tumors following CAR T cell treatment, we quantified Ki-67 staining of T cell and CAR T cell–treated tumors. Ki-67 staining was reduced with CAR T cell treatment in FaDu and SCC-47 tumors relative to T cell–treated tumors (Fig. 4F).

## Discussion

**FIGURE 5.**
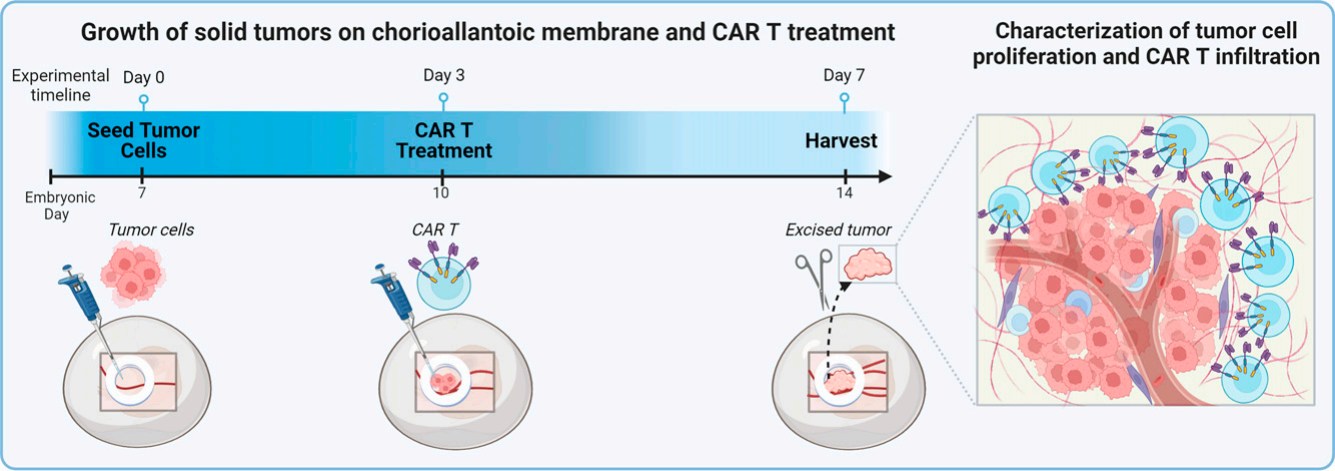
Visual abstract. Overview of chick CAM modeling of solid tumor treatment with CAR T. Engrafted tumor cells are treated three days following engraftment, and harvested seven days after engraftment to characterize T cell infiltration. The visual abstract was created using BioRender.com.

In this article, we use the chick CAM model to evaluate anti-tumor efficacy of CAR T cells against vascularized solid tumors (Fig. 5). Using clinically tested HER2-specific CAR T cells, we observed CAR T cell persistence in CAM-engrafted tumors and killing of cancer cells bearing the target Ag. We therefore suggest CAM tumor models as a promising candidate platform for preclinical testing of CAR T cell efficacy against head and neck cancer and other solid tumors.

HER2-specific CAR T cells were not equally effective in elimination of all HER2-expressing cell lines. This may reflect differences in characteristics such as aggressiveness of tumors from which the lines are derived such as the site of original tumor or mutation status, because both FaDu and SCC-47 lines express similar levels of HER2. Because SCC-47 expressed lower baseline levels of Ki-67 than FaDu tumors, this line may be relatively slower growing, resulting in a less dramatic treatment effect of CAR T cells when quantifying reduction in luminescence of viable cells after treatment.

Just as in the field of drug discovery, screening tools to evaluate CAR T cell efficacy are essential to select the ideal candidates for further preclinical and clinical testing. The progression of CAR T cells from development to clinical phase testing in solid tumors has been slow and met with many challenges. There is still no consensus on the best CAR construct to use for the treatment of solid tumors, and as the techniques for CAR design become more sophisticated, an increasing number of CAR T cell candidates are generated that require further testing.

The CAM system stands out as a potential bridge between in vitro and in vivo screening platforms: the vascularized solid tumors formed on the CAM are more structurally complex than spheroid and organoid cell cultures but less time-consuming and expensive than mouse models. Spheroid and organoid cell culture systems have increased in popularity as screening tools for drug treatment and discovery with a three-dimensional structure that is more intricate than monolayer cell culture yet faster to establish and cheaper than mouse models. These systems rapidly produce three-dimensional tumors in vitro (reviewed in Ref. 28), which in many ways accurately reflect patient tumor’s histological features (29). These systems can be readily manipulated to accommodate screening of numerous treatments. However, unlike xenografts grown in murine or chick CAM hosts, tumor spheroids and organoids are not vascularized. Additionally, organoid cultures often require the support of panels of growth factors or niche specific requirements (29), which can make studying expansion and survival of tumor cells difficult. In proceeding from in vitro or ex vivo to in vivo validation of CAR T cell anti-tumor efficacy, preclinical models have thus far been limited to cancer xenografts grown in immunocompromised mouse models. Most current preclinical studies of CAR T cells rely on transplanted human cancer cell line xenografts in immunocompromised murine hosts such as NOD/SCID/γc^−/−^ (NSG) mice that lack mature T cells, B cells, and functional NK cells (30, 31). These mice are expensive and require months of care over the course of an experiment. Furthermore, conventional mouse models do not naturally recapitulate the human immune system or immune infiltration into the tumor microenvironment (32, 33). Genetically engineered mouse models have recently been developed in which tumors arise spontaneously in transgenic mice with clinically relevant mutations (34–37) and evolve naturally with the murine host immune system. However, in genetically engineered mouse models, both tumor and immune cells are of murine origin, limiting their utility in assessing CAR T cell constructs intended for human use. Therefore, the potential value of the CAM model is its ability to balance the benefits of murine in vivo models such as solid, vascularized tumors in an immunodeficient host capable of accepting xenografts, with a relatively rapid, inexpensive, and high-throughput platform.

To enhance utility, further work could expand the ability of the CAM model to mimic the tumor immune microenvironment. Because immune responses to solid tumors are often limited by an immunosuppressive environment (38, 39), reconstitution of CAM tumors with T regulatory cells, myeloid-derived suppressor cells, cancer-associated fibroblasts, and other cells could be used to mimic the tumor milieu. This area of research could be greatly expanded to recreate many different aspects of the tumor immune microenvironment and enable screening of combination treatments and additional immunotherapies such as checkpoint blockade in the CAM model.

Interestingly, we noted that inoculated CAR T cells densely accumulated at the interface of tumor nests with the surrounding CAM, consistent with the immune-excluded phenotype of many solid tumors. This raises the possibility that CAM tumors may be appropriate models to test the efficacy of approaches to reverse immune exclusion and enhance T cell infiltration. As a related approach, because CAM-grown tumors are well vascularized by chick-derived vessels, it may also be possible to use techniques for intravascular injection of CAR T cells and other adoptive cellular therapeutics to better mimic the dynamics of CAR T cell trafficking to tumors. This model is also highly relevant for cancers such as oral cavity cancer, as was modeled in this article, for which techniques like intratumoral injection may be feasible.

The chick CAM model can also potentially be used as an individualized treatment model through implantation of patient-derived xenografts (PDXs). Our laboratory has developed strategies to more efficiently engraft PDX samples on the CAM, with the future goal of using CAM-engrafted PDXs for rapid therapeutic sensitivity testing (40). With the advancement of personalized medicine, the CAM could prove a useful tool in the development of tailored treatments based on an individual patient’s tumor sensitivities.

Taken together, our data suggest that the chicken egg CAM model is a potentially useful platform for rapid screening of anti-tumor efficacy of CAR T cells against solid tumors. We demonstrate herein that HER2-specific CAR T cells can persist in CAM tissue, traffic to tumors, and eliminate cancer cells grown on the CAM. We believe that the rapidity with which anti-tumor efficacy of CAR T cells can be evaluated on the CAM (in approximately 1 week), provides a strong rationale for further development of CAM-based tumors as a model for rapid screening of CAR T cells and other adoptive cellular therapies.
